# Left Ventricular Pseudoaneurysm: An Overview of Diagnosis and Management

**DOI:** 10.1177/2324709618792025

**Published:** 2018-08-02

**Authors:** Faisal Inayat, Ali Raza Ghani, Iqra Riaz, Nouman Safdar Ali, Usman Sarwar, Raphael Bonita, Hafeez Ul Hassan Virk

**Affiliations:** 1 Allama Iqbal Medical College, Lahore, Pakistan; 2 Abington Hospital—Jefferson Health, Abington, PA, USA; 3 Einstein Medical Center, Philadelphia, PA, USA

**Keywords:** left ventricular pseudoaneurysm, diagnosis, management

## Abstract

Left ventricular pseudoaneurysm is a rare but life-threatening disorder that is frequently reported secondary to myocardial infarction or cardiac surgery. In this article, we chronicle the case of a patient with no prior risk factors who presented with a 2-week history of nonexertional atypical left chest pain. Apical 2-chamber transthoracic echocardiography revealed an unexpected outpouching of basal inferoseptal wall of the left ventricle, which had a narrow neck and relatively wide apex. The patient was diagnosed with left ventricular pseudoaneurysm and medical therapy was initiated. He refused to undergo the surgical intervention and subsequently, he was discharged from the hospital in stable condition. This article illustrates that physicians should be vigilant for atypical presentations of left ventricular pseudoaneurysm, and a high index of suspicion should be maintained for this stealth killer while performing appropriate diagnostic imaging. Additionally, we review the currently available approaches to diagnosis and management in these patients.

## Introduction

Left ventricular pseudoaneurysm (LVP) is a rare but serious clinicopathologic entity. This outpouching is formed when cardiac rupture is contained by adherent pericardium or scar tissue, with no myocardial tissue. The clinical presentation can be nonspecific, including congestive heart failure, chest pain, dyspnea or arrhythmia leading to a delay in the diagnosis.^1^ Several case reports show its occurrence secondary to myocardial infarction (MI), cardiac surgery and interventions, infection, or trauma.^1,2^ This disease carries a significant risk of rupture due to a strong tendency to grow rapidly. Therefore, an early diagnosis and treatment are crucial in such patients.

Noninvasive imaging modalities, including echocardiography, computed tomography angiogram, and cardiac magnetic resonance imaging (CMRI), have been employed with a good diagnostic yield in patients with LVP.^3^ Medical therapy can be considered in asymptomatic patients with small-sized outpouching (<3 cm).^4^ However, a regular follow-up to monitor the dimensions is extremely important. Surgical intervention is recommended in symptomatic patients with giant aneurysms and those who carry considerable risk of rupture.^3^ Recently, a new therapeutic approach has been used in the form of percutaneous-device closure.^5^

In this article, we present a case of basal inferoseptal LVP. Although concurrent basal wall and septal involvement is extremely rare, this disorder should be kept in mind whenever patients present with nonspecific cardiopulmonary symptoms. Furthermore, we review the pertinent medical literature underscoring the importance of early diagnosis and appropriate management.

## Case Presentation

A 69-year-old African American male with a formidable medical history of paroxysmal atrial fibrillation (on amiodarone and warfarin), end-stage renal disease status post deceased-donor kidney transplant 2 months ago (on immunosuppressive therapy with mycophenolate, prednisone, and tacrolimus), hypertension, transient ischemic attack, right carotid artery stenosis status post carotid artery stent, and hyperlipidemia presented to our outpatient clinic for atypical left chest pain for 2 weeks. Pain was nonexertional, nonpositional, nonradiating, intermittent, and moderate in severity. In his last office visit after the kidney transplant, he was evaluated for light-headedness. He was found to be orthostatic hypotensive; therefore, blood pressure medications were adjusted that improved his dizziness. He endorsed good exercise tolerance. He self-medicated himself with antireflux medications, which helped his chest pain. The patient denied palpitations, shortness of breath, syncope, fever, chills, or headache. On admission, his vital signs indicated a regular pulse rate of 90 beats per minute and blood pressure of 110/70 mm Hg. The physical examination was unremarkable.

## Investigations

**Figure 1.**
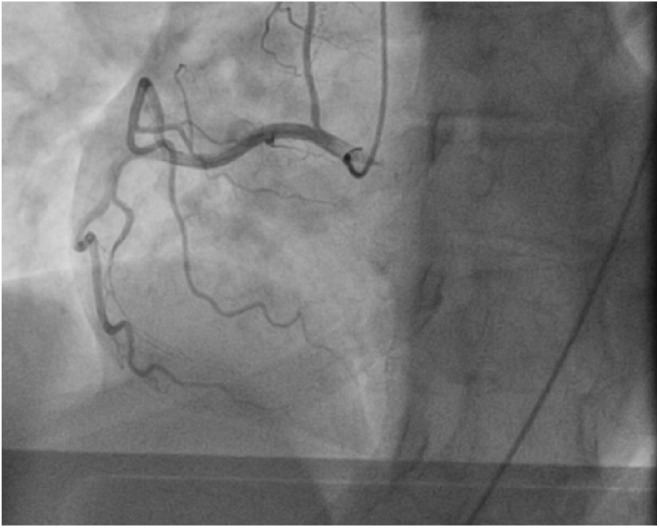
Left anterior oblique cranial view of right coronary artery system showing no obstruction.

**Figure 2.**
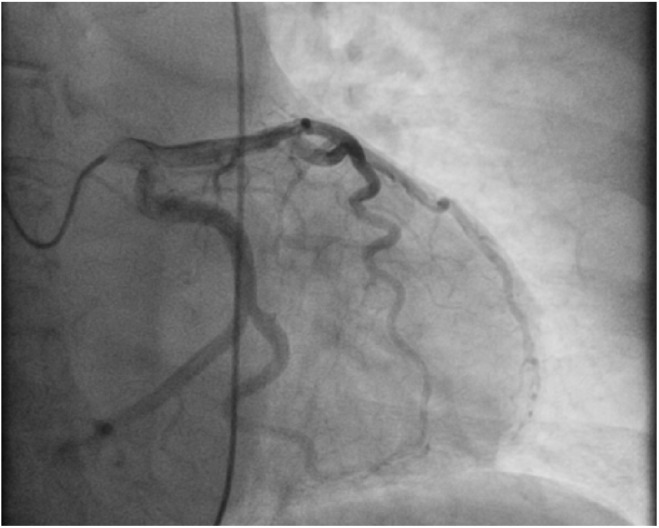
Left anterior oblique caudal view of left coronary artery ruling out stenosis.

**Figure 3.**
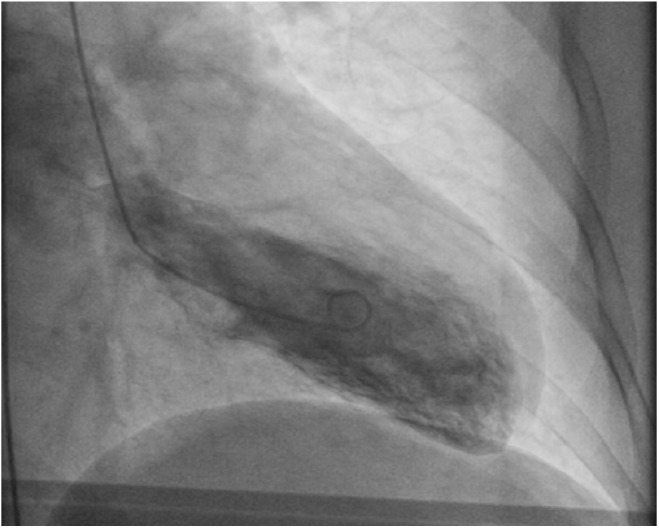
Left ventriculogram showed no wall motion abnormality.

**Figure 4.**
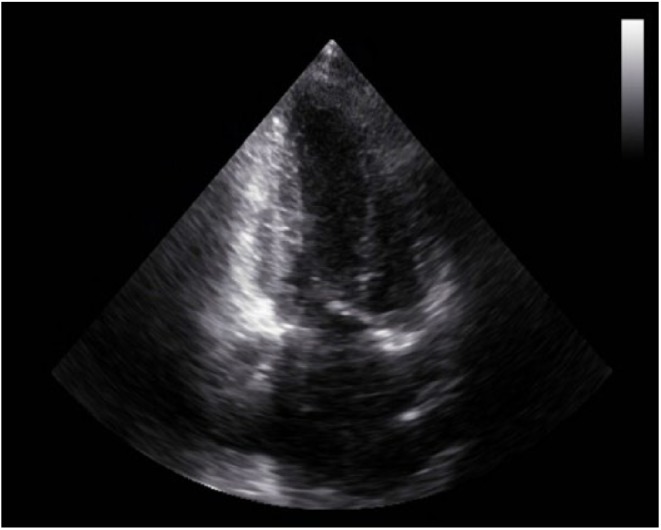
Baseline apical 2-chamber view of transthoracic echocardiogram 2 months ago showing normal basal to distal anterolateral, and inferoseptal walls of LV without any outpouching or aneurysm.

**Figure 5.**
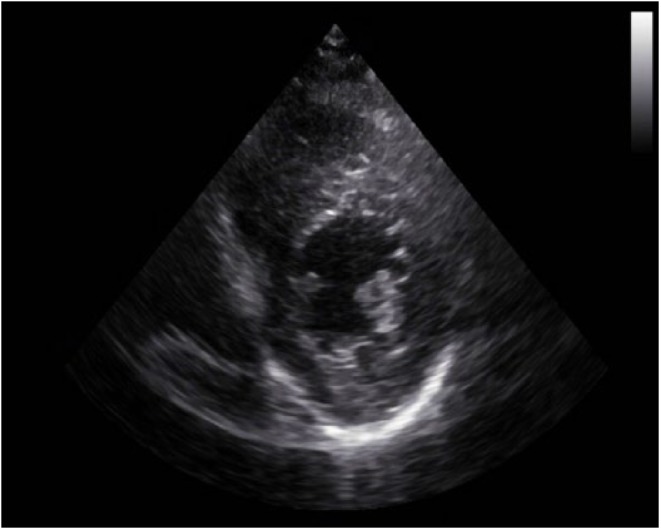
Parasternal short axis view of transthoracic echocardiogram across LV 2 months ago showing normal walls of LV without any outpouching or aneurysm.

**Figure 6.**
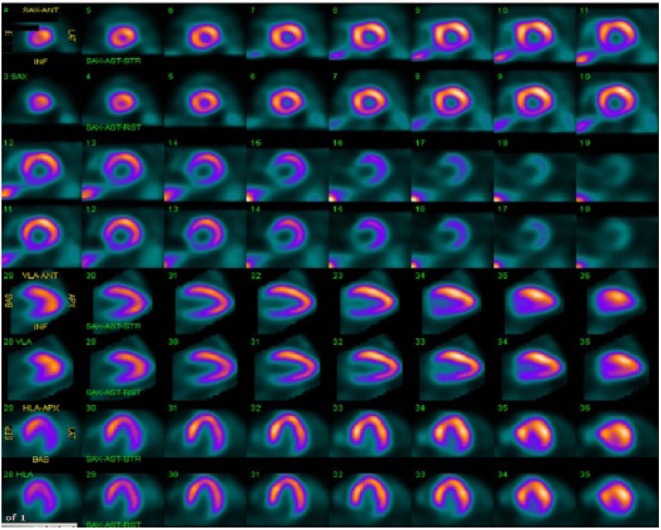
Nuclear scan performed 2 months ago showing no perfusion defect in inferoseptal wall.

**Figure 7.**
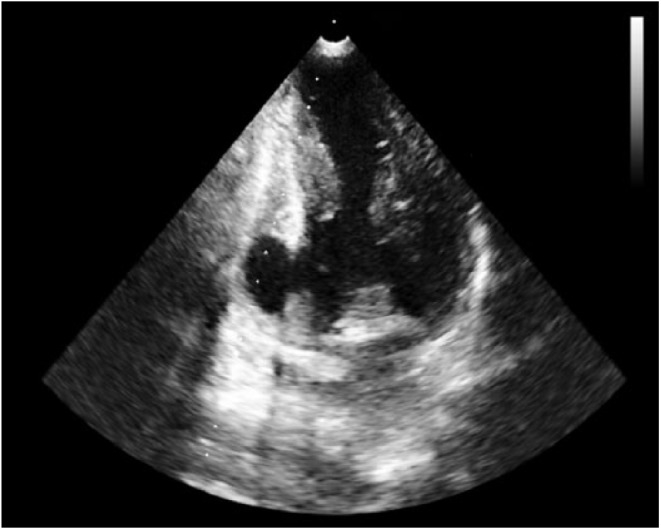
Apical 2-chamber view of transthoracic echocardiogram showing basal inferoseptal and inferior wall akinesis and thinning with pseudoaneurysmal formation (dimensions of pseudoaneurysm were 2.7 × 1.8 cm with neck width 1.6 cm) with dynamic contraction of the remaining walls.

**Figure 8.**
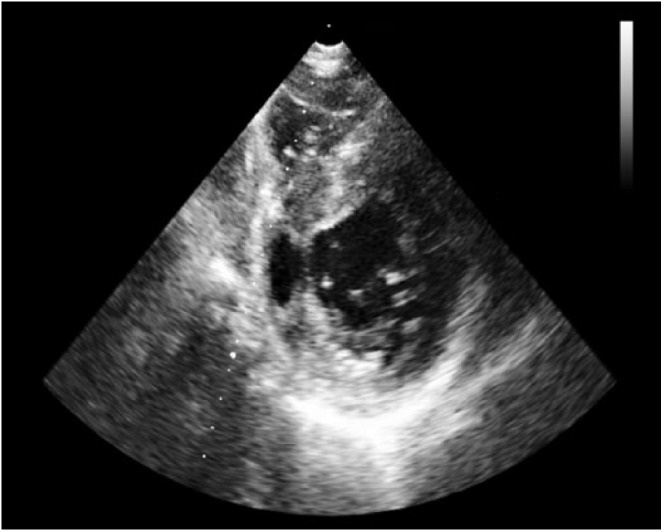
Parasternal short axis view of transthoracic echocardiogram across LV showing outpouching of inferoseptal and inferior wall.

A cardiac panel demonstrated normal troponin T and creatine kinase-MB levels. An electrocardiogram (ECG) showed sinus rhythm. He then underwent cardiac catheterization due to the refractory chest pain. Left anterior oblique cranial view of the right coronary artery system was negative for significant obstruction (Figure 1). Left anterior oblique caudal view of the left coronary artery also ruled out stenosis (Figure 2). Left ventriculogram showed no wall motion abnormality (Figure 3). Two months ago, ischemic workup was completed. His 2-dimensional transthoracic echocardiography showed normal basal to distal anterolateral and inferoseptal walls of the left ventricle (LV) without any outpouching or aneurysm (Figure 4). Parasternal short axis view across LV, 2 months ago, also revealed normal walls (Figure 5). Pharmacological nuclear stress test before kidney transplant was also inconclusive for any perfusion defect in the inferoseptal wall (Figure 6). Therefore, a plan was made to repeat the cardiac imaging and follow-up in few weeks. Therein, the apical 2-chamber view of transthoracic echocardiography showed contained rupture or pseudoaneurysm with a narrow neck of the basal inferoseptal wall of LV (Figure 7). Parasternal short axis view across LV showed outpouching of inferoseptal and inferior wall (Figure 8). This was a new finding when compared with the extensive cardiac imaging, performed 2 months ago.

## Differential Diagnosis

The differential diagnosis included chest wall pain, acute MI, panic disorder, pericarditis, pneumonia, pulmonary embolism, aortic dissection, LV aneurysm, and LV diverticulum. On the basis of clinical features, findings of echocardiography, and exclusion of other probable etiologies; he was diagnosed with LVP.

## Treatment

The patient was initiated on medical therapy with angiotensin-converting enzyme inhibitors for afterload reduction and β-blockers as an antianginal medication. The definitive surgical treatment was recommended and discussed with the patient. However, he refused to undergo the procedure.

## Outcome and Follow-up

He was discharged from the hospital in stable condition on medical therapy. The patient was educated about watchful monitoring of his condition and the importance of a regular follow-up. At the 6-month follow-up, repeat 2-dimensional transthoracic echocardiography ruled out progression of pseudoaneurysm, with the lesion showing same dimensions. He occasionally experienced intermittent atypical chest pain but was willing to stick to the medical therapy alone.

## Discussion

Ventricular pseudoaneurysm, unlike a true aneurysm, is enclosed by the pericardium, pericardial adhesions, or a thrombus. The outer layer of a true ventricular aneurysm always includes endocardium and myocardium with intact heart wall. Most of the LVPs develop after MI or cardiothoracic surgery. In a systematic literature review of 290 patients, MI (55%), surgery (33%), and trauma (7%) were the top 3 associations.^1^ LVPs carry a substantial risk of rupture, which is considerably higher than that of a true aneurysm. Therefore, an urgent management is of prime importance in these patients. Although new diagnostic techniques help diagnose LVPs early, the risk of rupture is still up to 30% to 45%.^1,6^ Surgical intervention can substantially decrease the risk of rupture. However, the progression of the disease and the risk of rupture in LVP patients with or without treatment are unclear as the findings are usually based on single-center case series, and it merits further investigation.^7^

The clinical presentation of patients with LVPs is varied. In a systematic literature review, 10% (n = 290) cases were completely asymptomatic.^1^ In another case series from Mayo Clinic, 48% (n = 52) cases were asymptomatic.^7^ Most common symptoms were chest pain and dyspnea. Sudden cardiac arrest, congestive cardiac failure, acute MI, syncope, tamponade, and embolism were other notable clinical presentations.^1,8^ A to-and-fro murmur related to mitral regurgitation could be heard in two-thirds of patients with LVPs. Similarly, positive findings on chest radiographs were observed in 50% of the cases. Ninety-five percent patients had nonspecific changes on ECG, with 20% having ST-segment elevations.^8^ However, the present case is unique in this regard as there were no abnormal changes on ECG.

The exact pathogenesis of LVP remains to be determined. These lesions mostly result when a small weak part of the cardiac wall, usually a transmural infarct ruptures, and a narrow orifice results over a relatively long period of time that connects the ventricular cavity with potential space beneath the pericardium.^9^ Blood flows to-and-fro through the narrow orifice and ultimately accumulation results under the umbrella of pericardium or pericardial adhesions. An outpouching over the affected area occurs due to this accumulation. As weak heart-wall protrusion is also observed in true ventricular aneurysms and clinical findings of both of these pathologies are nonspecific, differentiation is a diagnostic challenge. In a case series, it was shown that the site of a pseudoaneurysm is related to the etiology. The most common site for the development of a pseudoaneurysm was inferior or posterolateral wall after MI (in 82% of the MI patients).^7^ In 87% of the patients undergoing congenital heart surgery, right ventricular outflow tract pseudoaneurysm was seen.^7^ All patients undergoing mitral valve replacement and aortic valve replacement had the disease in the posterior subannular region of the mitral valve and the subaortic region, respectively.^7,9^ The patient in the current study did not have any of these abnormalities. Since pseudoaneurysm formation occurred without antecedent MI (normal coronary angiography), we assume that it was caused by an indolent infection after initiation of immunosuppressive therapy posttransplant, suppressed by antibiotics. This infection led to delayed pseudoaneurysm formation causing chest pain with which our patient had initially presented.

Early diagnosis carries paramount importance in the management and prognosis of patients with LVPs. Transthoracic echocardiography, transesophageal echocardiography, ventricular angiography, and CMRI have been utilized in the diagnosis and differentiation of these lesions. Previously, left ventricular angiography has been found to be the most conclusive and reliable method for diagnosing LVPs.^10^ Contrast ventriculography is the gold standard but it is seldom used. Transthoracic echocardiography and transesophageal echocardiography are 26% and 75% effective in making a definitive diagnosis, respectively.^11^ A narrow neck and a wide apex are the hallmarks of a pseudoaneurysm on echocardiography. CMRI is helpful in differentiating LVPs from true aneurysms, and it has a reported sensitivity of 100%.^12^ It also has a specificity of 83%, but it is underutilized due to the availability problems.^12^ In patients with LVPs, a sharp, delayed pericardial enhancement has been reported to be a frequent finding on CMRI.^13^ Multimodality imaging with multiple axes is recommended for the diagnosis of LVPs, which also helps in anatomic demarcation and curating a surgical plan for the patient.^14^

Medical treatment has been advocated in the past for patients with incidental finding of LVPs and for those having a higher risk of morbidity and mortality following surgery. Recently, preference of medical therapy in cases with chronic LVPs has been stressed, especially for lesions less than 3 mm in size.^4,15^ The main objective of the therapy is to decrease pseudoaneurysm enlargement. Furthermore, decreasing ventricular wall stress by decreasing afterload and decreasing the risk of thromboembolism are of considerable importance. With high surgical mortality (23% in the systemic review of 290 patients^1^ and 28% in a recent case series of 30 patients^6^), critical decision making in light of the benefits and detriments of surgical approach is warranted.

Percutaneous approaches can be utilized for smaller pseudoaneurysms, and it has a low risk of massive rupture, thromboembolism, and cardiac failure. Septal occluder devices have been used in closing the pseudoaneurysms via the percutaneous approach.^16^ In high-risk surgery patients and those requiring a redo cardiovascular surgery, percutaneous device closure of LVPs is preferable.^17,18^

Active surgical management of an LVP with patch closure or primary closure is a highly recommended first choice, especially in patients with the symptomatic and acute disease.^3^ It is reported that rupture risk in LVPs increases to 48% when managed with medical treatment alone.^19^ Although few reports showed uneventful 1-month-long medical management, it is unclear if surgical management should be pursued for the chronic LVPs that are found incidentally.^7^ Post-MI LVPs benefit the most from the surgical closure of the pseudoaneurysms, if the surgical intervention is performed within 2 to 3 months of the episode.^20^ However, future research should aim to stratify the risks and indications of surgery in patients with LVP.

LVPs are rare and an estimated incidence post-MI is stated to be less than 2%.^10^ An earlier report suggests an incidence of as low as 0.2% of the MI cases.^21^ Although basal and inferior wall LVPs have been previously reported, basal and inferoseptal involvement, as in the present case, is extremely rare.^2,16^ The present case is unique in a sense that there was no prior history of major risk factors, including MI, surgery, trauma, infective endocarditis, or other rare conditions, that have been reportedly associated with LVPs. Moreover, at the time of the presentation, there were no abnormal ECG changes. This article calls for a lower threshold for multiaxial transthoracic echocardiography in patients with ischemic chest pain. It is a challenge to diagnose these patients and extreme vigilance on part of the concerned physicians is particularly warranted.

## Learning Points

1. LVP results from a cardiac-free wall rupture contained by adherent pericardium or scar tissue without any involvement of myocardium or endocardium. It frequently occurs in the setting of MI, surgery, trauma, and endocarditis.
2. A high index of clinical suspicion is required for early diagnosis and appropriate management.
3. Although patients with LVP can be completely asymptomatic, most symptomatic patients will report symptoms of heart failure, dyspnea, or chest pain. Therefore, physicians should be vigilant for the atypical initial clinical presentations of LVPs.
4. Cardiac catheterization with left ventriculogram is the gold standard for diagnosis. Transthoracic echocardiography can be used as an initial test in patients with suspicion of LVP. CMRI has been increasingly used as noninvasive diagnostic technique.
5. Medical therapy has been employed in selected patients; however, surgery is the treatment of choice. Untreated lesions carry a significantly high risk of rupture that leads to high morbidity and mortality in such patients.
